# Behavior of Human Osteoblast Cells Cultured on Titanium Discs in Relation to Surface Roughness and Presence of Melatonin

**DOI:** 10.3390/ijms18040823

**Published:** 2017-04-13

**Authors:** M. Fernanda Sola-Ruiz, Carolina Perez-Martinez, Carlos Labaig-Rueda, Carmen Carda, J. Javier Martín De Llano

**Affiliations:** 1 Department of Stomatology, Faculty of Medicine and Dentistry, University of Valencia, 46010 Valencia, Spain; carolinepm@hotmail.com (C.P.-M.); carlos.labaig@uv.es (C.L.-R.); 2 Department of Pathology and Health Research Institute of the Hospital Clínico (INCLIVA), Faculty of Medicine and Dentistry, University of Valencia, 46010 Valencia, Spain; carmen.carda@uv.es (C.C.); j.javier.martin@uv.es (J.J.M.D.L.)

**Keywords:** osteoblasts, titanium, roughness, melatonin, proliferation, differentiation, mRNA, PHEX, dental implants

## Abstract

The aim of this work was to observe the behavior of osteoblast cells cultured in vitro on titanium discs in relation to disc surface roughness and the addition of melatonin to the culture medium. MG63 osteoblast cells were cultivated on 120 Grade 5 Ti divided into three groups: Group E, treated with dual acid etch; Group EP, treated with dual acid etch and calcium phosphate; and Group M, machined. Surface roughness was examined under a laser scanning confocal microscope (CLSM) and scanning electron microscopy (SEM). The proliferation and morphology of cells were determined under fluorescence microscopy and SEM. Messenger ribonucleic acid (mRNA) of different genes related to osteoblastic differentiation was quantified by means of real-time quantitative polymerase chain reaction (RT-PCR) assay. The greatest surface roughness was found in Group EP (Ra 0.354 µm), followed by Group E (Ra 0.266 µm), and Group M (Ra 0.131 µm), with statistically significant differences between the groups (*p* < 0.001). In the presence of melatonin a trend to a higher cell proliferation was observed in all groups although significant differences were only found in Group M (*p* = 0.0079). Among the genes studied, a significant increase in phosphate-regulating neutral endopeptidase, X-linked (PHEX) expression was observed in cells cultured on EP discs. The addition of melatonin increased osteoblast cell proliferation and differentiation, and may favor the osseointegration of dental implants.

## 1. Introduction

The clinical success of dental implants depends on osseointegration, the direct structural and functional connection between living bone and the implant surface [1]. Titanium (Ti) is the material of choice for fabricating dental implants due to its excellent biocompatibility, its mechanical properties, and resistance to corrosion [2,3]. The addition of alloy elements makes it possible to modify and improve the structural and surface properties of Ti; in particular, the Ti6Al4V alloy, known as Grade 5 Ti, is one of the most widely used and has been shown to produce positive osseointegration outcomes in in vitro [4,5,6,7] and in vivo studies [8,9,10]. In addition to the properties of the material itself, its surface characteristics—both topographical (surface roughness) and physico-chemical—determine the molecular interactions produced in the first instance between proteins and the implant surface [11].

In this context, current research centers on managing the different properties derived from surface characteristics in order to improve the relationship between implant and living tissue, and maximize osseointegration in both the short and long term. Among the surface treatments available, acid etching is one of the most widely used producing a microtexture on the titanium surface without leaving any residue. This can be performed with a single acid [11] or a combination of acids, known as dual acid etch [12,13,14,15].

An implant’s surface roughness is an influential factor, affecting the absorption of proteins, adhesion, the proliferation of both osteoblasts and osteoclasts, and the mineralization of the bone tissues’ extracellular matrix. Different authors have obtained different, sometimes conflicting, roughness results, which may be due to the different methodologies employed to quantify Ra (and other differences) [14,16,17,18,19,20].

Surface roughness can be assessed qualitatively using scanning electron microscopy (SEM) or quantitatively by means of techniques such as contact profilometry [4,21]. Although with the latter technique measurement may be affected by distortion of the test surface as the device’s stylus moves over it. For this reason, contactless scanning techniques are more accurate. These include interferometry or confocal laser scanning microscopy (CLSM), which provide a higher-resolution register than contact profilometry [5,18,22]. Optical profilometry provides high resolution but requires a longer time to capture three-dimensional profiles [7,16,18,19,22].

Different implant coating materials can be applied to the surfaces; calcium phosphate is one of the most important, phosphates being the main components of the human body’s hard tissues, including bone and cartilage, dental enamel, and dentin [23]. Implant coating is performed by means of the discrete crystalline deposition process (DCD^®^, Biomet-3i, Paterna, Spain), which coats the Ti surfaces with nanometric crystals [8].

Other authors such as Habibovic have used a process of biomimetic precipitation with a calcium phosphate solution applied at a pH and temperature similar to the human body [24]. Chen used a method of electrophoretic deposition to coat Ti discs with calcium phosphate [25]. The classic deposition method uses particle projection, but this has been associated with clinical problems including delamination of the coating [26,27,28].

Short term in vitro studies that cultivate osteoblasts on different substrates can provide information about the main reaction events between implants and osteoprogenitor cells, as well as helping to identify and select those substrates that promote rapid healing and regeneration of the host bone, and in the last instance, interfere in long-term responses [3,4].

One of the most widely used cells types for in vitro culture on Ti discs is the commercially available cell line MG63 [6,8,16,17,29,30,31,32,33]. These cells have osteoblastic characteristics including high levels of alkaline phosphatase (ALP) activity and osteocalcin production during their differentiation [14,16]. Although these cells are obtained from human osteosarcoma, it has been shown that they have characteristics comparable to human osteoblasts. Other authors have used other cell lines with osteoblastic characteristics, such as MC3T3 [4].

Melatonin is a hormone synthesized and secreted by the pineal gland, which was first applied topically to implants [34]. Melatonin has a capacity to stimulate bone formation and inhibit resorption. It helps bone development through the inhibition of osteoclast differentiation [35,36] and by stimulating osteoblast differentiation [37,38]. There is also some controversy as to the optimal dose of melatonin added to cell culture medium [37,39]. Some authors have concluded that the most effective concentration is 50 µM [40].

In bone formation, melatonin stimulates the proliferation and synthesis of type I collagen in human osteoblasts cultured in vitro [40]. In preosteoblastic cultures, it has been seen that the gene expression of bone sialoprotein and other proteic bone markers, including alkaline phosphatase (ALP) and osteocalcin, accelerate differentiation dose-dependently [39].

It has been reported that topical application of melatonin at a dose of between 5 and 500 μM reduced mRNA of receptor activator of nuclear factor-kappa B (RANK) dose-dependently, and increased osteoprotegerin (OPG) levels and mRNA in MC3T3-E1 preosteoblast cell lines, inhibiting osteoclastogenesis by blocking preosteoclast differentiation [41]. The expression of diverse genes has been associated with the process of osteoblast cell defferentation; these include those that encode alkaline phosphatase (*ALPL*), collagen type I, alpha 1 chain (*COLIA1*), osteocalcin/bone gamma-carboxyglutamic acid-containing protein (*BGLAP*), phosphate-regulating neutral endopeptidase, X-linked (*PHEX*), and osteonectin/secreted protein acidic and cysteine rich (*SPARC*) [30]. Therefore, the quantification of the expression of these genes by means of real-time quantitative PCR makes it possible to determine the influence of diverse factors (such as cell culture surface characteristics or the composition of the culture medium) on this process [6,13,15,30,42].

Alkaline phosphatase (ALP) is a good indicator of osteoblast differentiation, which leads to extracellular matrix formation, maturation and mineralization [6]. The enzyme ALP is produced by osteoblasts and allows extracellular matrix mineralization by increasing the concentration of phosphate ions and inhibiting phosphoric ester action [4]. The gene *COL1A1* encodes the alpha 1chain of collagen type I, a protein that represents 90% of osteoid substance in the mineralized extracellular matrix of bone tissue.

Osteocalcin, a protein with γ-carboxyglutamic acid residues, is a late marker of osteoblast phenotype due to its presence in mature extracellular matrix. It has a high affinity with, and attaches to, calcium and hydroxyapatite, being a modulator of hydroxyapatite crystals [5,43]. The transmembrane endopeptidase that encodes the *PHEX* gene belongs to the type II integral membrane zinc-dependent endopeptidase family. This protein is involved in bone and dentin mineralization through phosphate reabsorption regulation [44,45]. Osteonectin is a glucoprotein produced by osteoblasts during bone formation. It permits the anchorage of bone cells in the bone matrix required to perform its mineralization.

Glyceraldehyde 3-phosphate dehydrogenase (*GAPDH*) is one of the most widely used housekeeping genes for managing and normalizing the expression of other genes [7,15,17,20,30,46].

On the basis of the above, the present work proposes the following hypotheses:

1. Surface treatment influences the surface roughness (Ra) of titanium discs;
2. Melatonin increases the adhesion and proliferation of human osteoblast cells on titanium surfaces;
3. Melatonin influences the differentiation of human osteoblast cells on titanium surfaces.

The objectives of this study were as follow:

1. To study the surface topography and roughness (Ra) of machined Grade 5 Ti, treated with dual acid etching or dual acid etching plus calcium phosphate coating, prior to cell culture;
2. To analyze the influence of the addition of melatonin to the cell culture media on the adhesion, proliferation, morphology, and differentiation of human osteoblast cells cultured on Grade 5 Ti with different surface treatments.

The conclusions obtained were:

1. The surface roughness of Grade 5 Ti discs (Ti6Al4V) varied according to the surface treatment applied. Machined Ti discs presented the least roughness (Group M), followed by those treated with dual acid etch (Group E), and dual acid etch with calcium phosphate coating (Group EP), which presented the highest roughness (Ra) values, with statistically significant differences between groups;
2a. The presence of 50 µM melatonin in cell culture media resulted in statistically significant differences in cell proliferation between groups, Group M presenting the greatest, followed by Group E, and EP when cells were cultured for 72 h.
  Additionally, the only statistically significant difference was between Group M with melatonin and Group M without melatonin.
2b. The addition of melatonin increased the relative expression of the *PHEX* gene in Group EP after 1 week culture, with statistically significant differences in comparison with Group M (*p* < 0.05) and Group E (*p* < 0.05).

After five weeks of cell culture, *PHEX* showed the highest expression in the EP group, with statistically significant differences in comparison with group E. Melatonin increased *PHEX* gene expression in all groups after five weeks culture in relation to its homologues without melatonin, favoring cell differentiation.

## 2. Results and Discussion

### 2.1. Surface Roughness of Ti Discs

**Figure 1:**
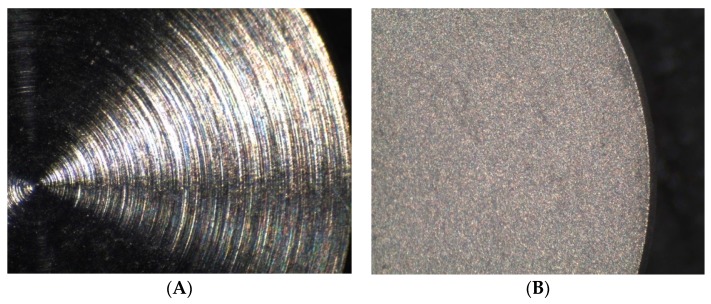
Microphotographs taken with an optical microscope (35×). (**A**) Image of a Group M disc; (**B**) Image of a Group E disc.

Images captured by optical microscope at relatively low magnification clearly showed differences between treated surfaces (Groups E and EP) and machined disc surface (Group M) (Figure 1A,B). Machined discs had a shiny surface on which the regular concentric lines of the disc machining process were clearly visible. Treated disc surfaces (Groups E and EP) showed a matt surface of sandy appearance, characteristic of the acid etching process.

**Figure 2:**
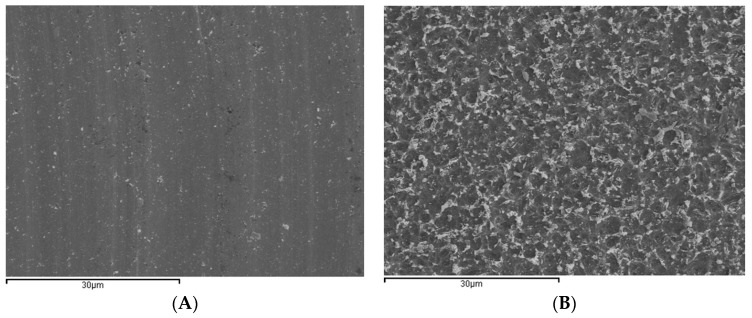
Microphotographs captured by scanning electron microscopy (SEM) (2000×). (**A**) Image of a Group M disc showing the lines produced by machining; (**B**) Image of a disc in Group E showing characteristic microtexture of pits and hollows.

Microphotographs captured by SEM again show the lines produced by machining on Group M discs, while Group E acid etched discs and Group EP acid etch + calcium phosphate present characteristic microtexturing with irregular pits and hollows (Figure 2A,B).

**Figure 3:**
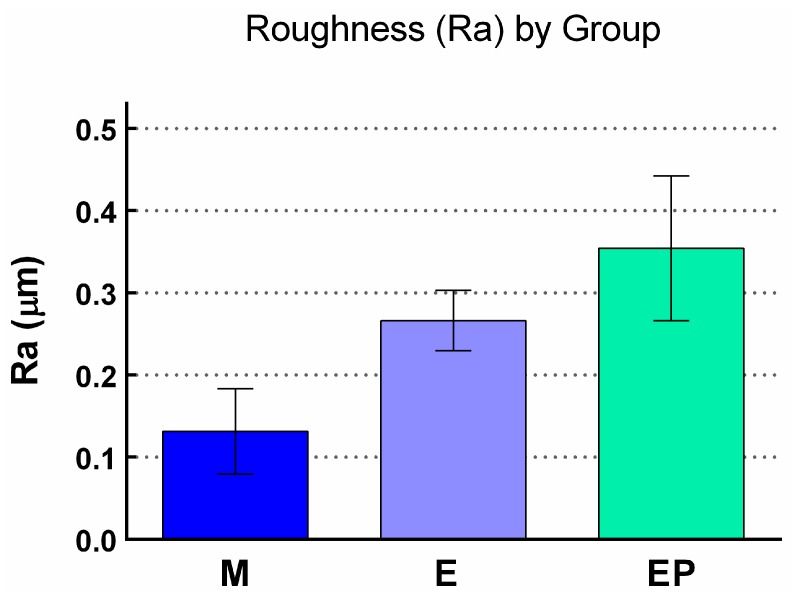
Box plot of roughness (Ra) in different study groups (mean ± SD).

After measuring the roughness of the discs using confocal laser scanning microscopy (CLSM), three random measurements were taken on each of the 120 discs, obtaining a total of 360 Ra values. Mean roughness (Ra) showed that Group EP exhibited the greatest roughness with a Ra of 0.354 ± 0.088 µm, followed by Group E (0.266 ± 0.037 µm), and lastly Group M (0.131 ± 0052 µm). (Figure 3, Table 1). Differences between the three groups were statistically significant (*p* < 0.001), according to an ANOVA simple linear regression model *F* test.

Table 1 provides the roughness data obtained for the three study groups.

When results were analyzed comparing groups in pairs it was found that mean roughness in Group E was significantly greater than the control group (Group M) (*p* < 0.001), mean roughness in Group EP was significantly greater than the control group (Group M) (*p* < 0.001), and mean roughness in Group EP was significantly greater than Group E (*p* < 0.001, applying Tamhane’s *T*2 test).

The results confirm that machined discs without any additional surface treatment, presented lower Ra values than treated discs, regardless of the treatment applied, these results being similar to those published by other authors using sand-blasting [16,17], acid etch [14], and coating with different materials [17,19,20]. These findings validate the first hypothesis: surface treatment influences Grade 5 Ti surface roughness.

### 2.2. Analysis of Cell Adhesion (24 h) and Proliferation (72 h)

**Figure 4:**
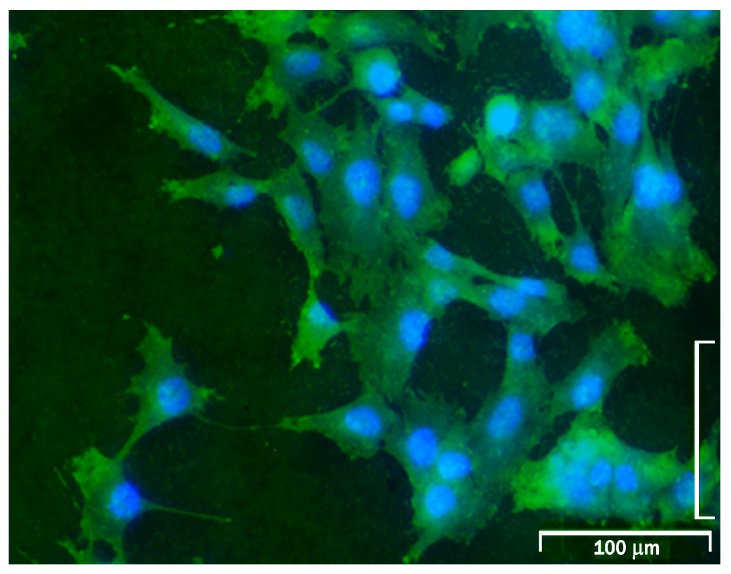
Microphotograph of a Group E disc showing the blue fluorescence of cell nuclei stained with 4′,6-diamidino-2-phenylindole (DAPI) and the green fluorescence of cell cytoplasm components stained with eosin.

Cell density (cells/mm^2^) on the disc surfaces was calculated by determining the total number of nuclei present on the whole surface of each disc. The total surface was digitally reconstructed by merging overlapping images captured with a fluorescence microscope, like the image shown in Figure 4.

Table 2 shows mean cell density values obtained in the three study groups: M (machined), E (dual acid etched) and EP (dual acid etched and calcium phosphate coating) according to culture time and the addition of melatonin to the culture medium.

**Figure 5:**
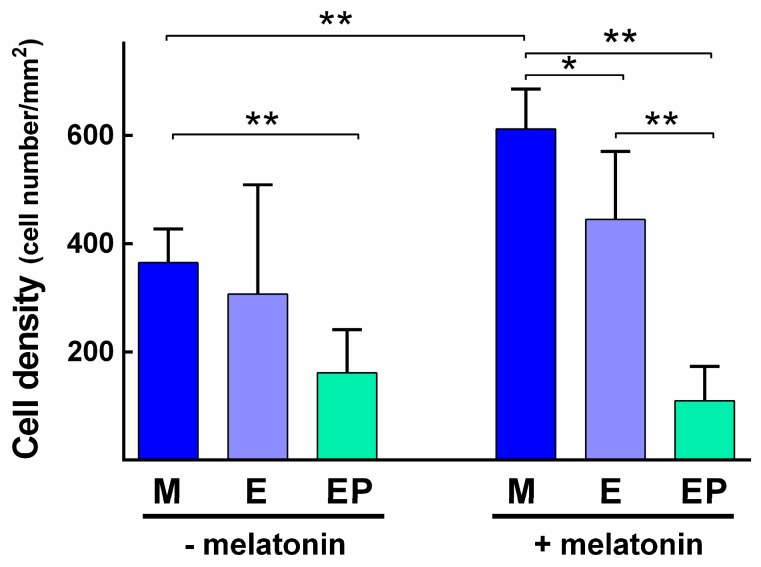
Cell density (mean and SD) of cells grown on discs of the three experimental groups after 72 h of cell culture without or with melatonin added to the cell culture media. Statistically significant differences between groups are shown (*, *p* < 0.05; **, *p* < 0.01.).

Twenty-four hours after cell seeding and when melatonin was not added to the culture medium, no statistically significant differences were found in cell density between the three experimental groups. After 72 h of cell culture, the greatest proliferation was found in Group M, followed by Group E and lastly Group EP, with statistically significant differences between Groups M and EP (*p* = 0.0079) (Figure 5).

Several studies have shown that Ti surface roughness is directly associated with osteoblast adhesion and proliferation and therefore with the subsequent development of mineralized tissue at the implant interface. Various authors corroborate the present results, observing greater cell proliferation on the Ti surface as roughness decreases [13,15,17,43]. Thus, this EP Group with a hydroxyapatite coating showed the lowest values with statistically significant differences, as observed by other authors, [47]. Nevertheless, other studies, like that from Chen et al, observed greater cell proliferation on the roughest discs [25].

Twenty-four hours after cell seeding and in the presence of melatonin in the culture medium, no significant differences were found between the groups. After 72 h of cell culture, statistically significant differences in cell proliferation were found between groups (M vs. E, *p* = 0.0317; M vs. EP, *p* = 0.0079; E vs. EP, *p* = 0.0079), Group M presenting the greatest, followed by Groups E, and EP (Figure 5). When comparing each group with and without melatonin added to the cell culture media, the only statistically significant difference was between Group M with melatonin and Group M without melatonin (*p* = 0.0079) after 72 h of cell culture (Figure 5).

Melatonin can influence cell proliferation, either inhibiting or favoring it depending on the cell type, the melatonin concentration, and the culture time [48,49]. The results described herein after 72 h of cell culture show that the addition of melatonin to the culture medium at a concentration of 50 µM boosted cell proliferation.

Several studies concur with the present findings. Nakade used human mandibular cells (HOB-M) and human osteoblast cell line cells (SV-HFO); cells were cultured without melatonin (control group) and at different concentrations of melatonin (5, 10, 50, and 100 µM) in the culture medium. The dose that produced the greatest proliferation of both cell types after 72 h culture was 50 µM, although variations were observed depending on cell type [40]. Satomura cultured human osteoblasts harvested from mandibular bone with the addition of melatonin in doses between 1 and 200 µM. Proliferation was evaluated after five days, obtaining higher cell proliferation levels at doses greater than 50 µM, with statistically significant differences [42]. In the same way, Zhang cultured human mesenchymal stem cells (hMSCs) with melatonin present in the cell culture media (at concentrations of 0.01, 1, and 100 µM) and without melatonin. It was concluded that although melatonin generally increased proliferation, it did not appear to affect hMSCS proliferation [39]. There is likely an additive effect, so melatonin induces a higher proliferation when cells are growing on a smoother surface.

The present findings validated the second hypothesis that melatonin at a concentration of 50 µM added to the culture medium favors or increases the adhesion and proliferation of human osteoblast cells on titanium surfaces.

### 2.3. Morphological Analysis with Scanning Electron Microscopy (SEM)

**Figure 6:**
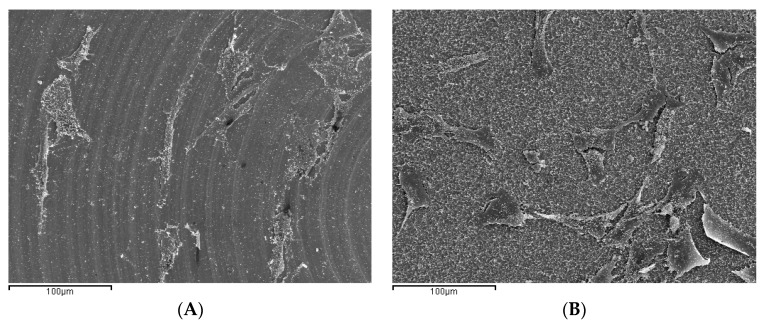
Scanning electron micrograph of MG63 cells cultured on titanium disc surfaces (350×). (**A**) Group M disc showing osteoblast adhered over the surface following the concentric machining lines; (**B**) Group E disc where cells were distributed randomly over the whole surface.

After 24 h of cell culture, osteoblasts were actively adhered to both treated and machined Ti disc surfaces. Microphotographs captured by SEM at 350× show differences between surfaces and how the surfaces affected cell behavior. Completely different adhesion patterns were observed between machined or treated surfaces. On Group M discs, osteoblasts adopted a concentric dispersion following the machined lines, while on treated discs they were dispersed randomly over the surface (Figure 6A,B).

**Figure 7:**
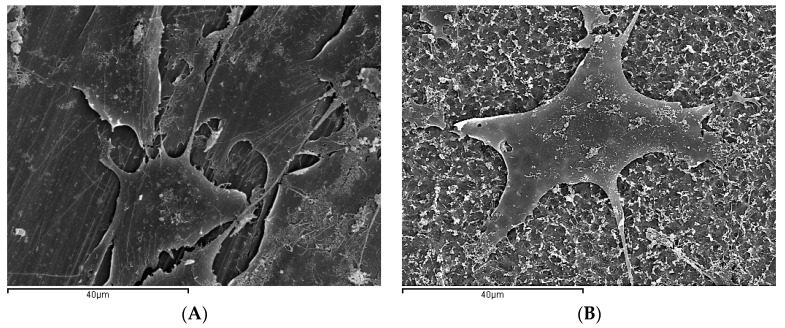
Scanning electron microphotographs of MG63 cells cultured of titanium disc surfaces (2000×): (**A**) Group M disc cells of flat morphology could be seen on the machined discs in close contact with the surface with cytoplasmic prolongations and filopodia; (**B**) Group E cells were more rounded or cuboid, more regular and with shorter prolongations.

At greater magnifications (2000×) (Figure 7A,B) cells of flat morphology could be seen on the machined discs in close contact with the surface with cytoplasmic prolongations and filopodia, which help them to adhere more effectively to the surface and move over it, as observed by other authors [15,25,50].

According to the results published in different works, the morphology of cells grown on discs with different surface treatments differ depending on the treatment applied to the disc. In the present work, the cells were more rounded or cuboid, more regular and with shorter prolongations, as shown by other authors, [15,51]. However, authors described cells as having irregular edges and large numbers of filopodia or pseudopodia [16,52]. In the present study, it was not possible to identify clear differences in cell morphology between the different surface treatments. To do so, it would be necessary to perform quantitative morphometric studies, measuring the cell area or roundness in order to determine whether there are real differences in morphology between cells grown on acid etched discs and acid etched discs coated with calcium phosphate.

### 2.4. Quantification of Specific mRNA

**Figure 8:**
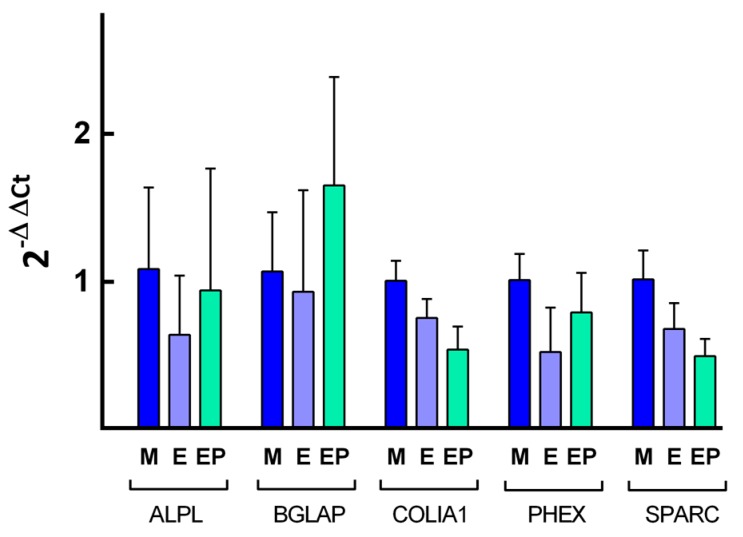
Quantification of the relative expression of the indicated genes after one week of cell culture without melatonin of the indicated genes. The mean *F*c and SD value are represented for the different genes, taking the average expression of cells grown on Group M discs as control.

**Figure 9:**
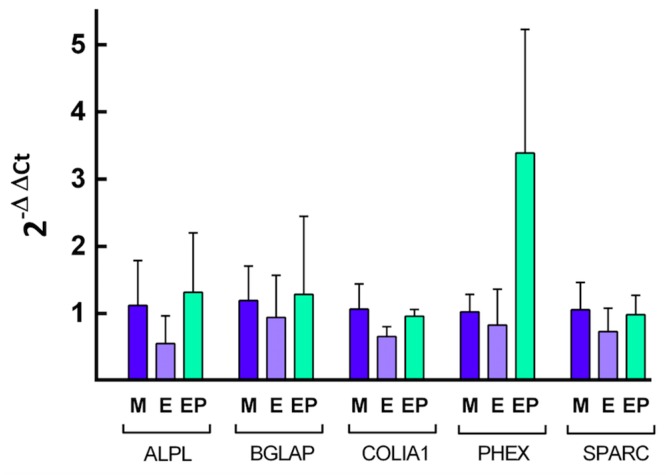
Quantification of the relative expression of the indicated genes after one week of cell culture with melatonin added to the culture media. The mean *F*c and SD values are represented for the different genes, taking the average expression of cells grown on Group M discs as control.

**Figure 10:**
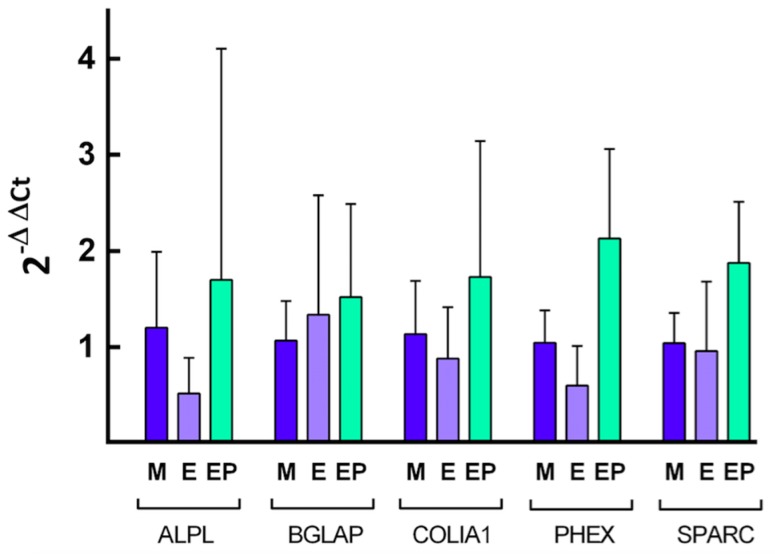
Quantification of the relative expression of the indicated genes after five weeks of cell culture without melatonin of the indicated genes. The mean *F*c and SD value are represented for the different genes, taking the average expression of cells grown on Group M discs as control.

**Figure 11:**
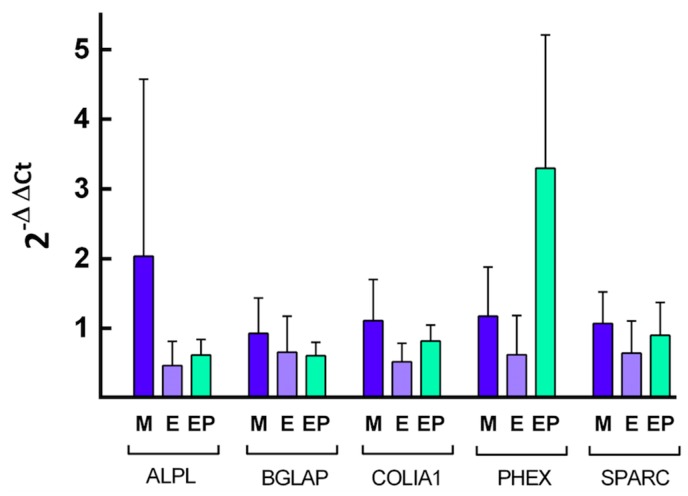
Quantification of the relative expression of the indicated genes after five weeks of cell culture with melatonin added to culture media. The mean *F*c and SD values are represented for the different genes, taking the average expression of cells grown on Group M discs as control.

Quantification of relative expression of different genes related to osteoblast differentiation was performed by calculating the value of 2^−ΔΔ*C*t^, using the endogenous expression of the gene *GAPDH* as a control, or so-called “housekeeping” gene, to normalize expression levels. The Figure 8, Figure 9, Figure 10 and Figure 11 show the mean value of *Fc* (fold change) of selected gene expression after one and five weeks of MG63 cell culture on discs of the three experimental groups with and without melatonin added to the culture media.

The results were analyzed applying the non-parametric Mann-Whitney test, comparing the variation in relative gene expression of the different genes between pairs of Ti disc experimental groups. Comparing each gene expression level among the study groups after one week of cell culture without melatonin added to cell culture media, no clear pattern of gene expression was observed (Figure 8). Melatonin increased the relative expression of the gene *PHEX* in Group EP, which showed the greatest relative expression with statistically significant differences in comparison with Group M (*p* < 0.05) and Group E (*p* < 0.05) expression level (Figure 9).

After five weeks of cell culture without melatonin added to the cell culture medium (Figure 10), cells in Group EP showed the greatest relative expression for all the genes analyzed, the highest being that from *PHEX* gene, with statistically significant differences in comparison with Group E (*p* < 0.001). When cells were cultivated for five weeks with melatonin added to the medium (Figure 11), the greatest relative gene expression was for *PHEX* gene in the EP Group, with statistically significant differences in comparison with Group E (*p* < 0.05) and difference with Group M although this did not reach statistical significance (*p* = 0.056). These findings validate the third hypothesis: melatonin influences human osteoblast differentiation cultivated on Ti surfaces.

Various studies corroborate these results, including that of Satomura in a study using human osteoblasts cultured with melatonin at concentrations between 1 and 200 µM who evaluated the quantity of mRNA of *COLIA 1*, *OCN*, *OPN*, and *BSP*. The addition of melatonin increased gene expression in the same way as the present study after one week of cell culture [42]. Zhang described higher levels of *ALP*, *OPN*, and *OCN* gene expression after 12 days of cell culture when melatonin was present in the cell culture media, in dose-dependently [39]. A study by Son, using MC3T3 cells cultured in media containing melatonin at concentrations between 10 and 250 µM. quantified gene expression of *ALP*, *COLIA1*, *OCN*, and *OSX* after 1, 3, 7, and 14 days of cell culture showed that gene expression increased in dose and time-dependent manner [46]. However, a study by Nakade using human osteoblast cells and the addition of melatonin to the cell culture media at concentration between 5 and 100 µM, measured different markers of osteoblast differentiation after four days of cell culture. A significant increase was found for *COLIA1* production in samples cultured in 50 and 100 µM melatonin, but no increase in ALP activity or osteocalcin production were observed [40].

In general, the published studies observations coincide with the present and with one another results, showing higher mRNA or proteins levels from genes related to osteoblast differentiation and mineralization, in cell cultured on Ti discs with greater surface roughness [13,16,17,30,43]. Nevertheless, comparison of the present results with other studies is made difficult because of variations in cell culture times, genes and proteins quantified, quantification methods used, the surfaces on which cells were cultured, as well as the cell lines used [15,51,53].

## 3. Materials and Methods

The study used 120 discs of 6 mm diameter and 2 mm thickness made from Grade 5 Ti, an alloy of titanium, aluminum, vanadium (Ti6Al4V) used for fabricating dental implants, supplied by Biomet 3i^®^ (Paterna, Spain). The discs were distributed randomly into three groups of 40 discs each: Group E, discs treated with dual acid etch (hydrochloric acid and sulfuric acid treatment); Group EP, discs treated with dual acid etch and with deposition of calcium phosphate particles; and Group M (control group), machined discs without any additional surface treatment.

All the discs were milled and machined, sectioning Ti grade 5 rods with an automatic lathe (Star Micronics^®^ Co., Ltd., Otelfingen, Switzerland). Afterward, these were washed using industrial ultrasound equipment (ATU Ultrasonidos^®^, Valencia, Spain) in a soapy solution at 60 °C to eliminate traces of oil or other possible residue derived from the machining process without damaging the surfaces. Discs were then dried in a forced convection oven (JP Selecta^®^, Barcelona, Spain).

Forty discs were separated randomly to form Group M (control group) and the remaining 80 discs were etched with sulfuric acid for 1 min. After a quick wash in distilled water, they were etched a second time with hydrochloric acid. The discs were immersed in a bicarbonate solution to neutralize the acid action and were washed with distilled water. Then the discs were passivated with nitric acid to obtain homogenous surfaces and were washed again in distilled water to complete the dual acid etch process.

Forty discs were separated to form Group E and the remaining 40 discs (Group EP) underwent calcium phosphate coating using hydroxyapatite [Ca_10_(PO_4_)_6_(OH)_2_], by means of a calcium phosphate suspension in 99.5% ethanol. The 40 discs from each experimental group were placed in individual containers and numbered from 1 to 40, so that each one could be monitored individually at any moment during the study.

The discs were examined under an optical microscope (Carl Zeiss^®^, OPMI Pico Dental, Jena, Germany) and a scanning electron microscope (SEM) (Jeol JSM 6300^®^, Oxford Instruments Ltd., Abingdon, UK). Mean surface roughness (Ra) was evaluated for all 120 discs using a confocal laser scanning microscope (CLSM) (MicroSpy Topo^®^, Fries Research & Technology GmbH, Bergisch Gladbach, Germany). Three Ra measurements per disc were taken making a total of 360 measurements. Data were processed with FRT Mark III software (Fries Research & Technology GmbH^®^, Bergisch Gladbach, Germany) for evaluation and collation. Then all discs were washed and placed in their individual packaging and sterilized by means of irradiation with gamma rays (Steris Corp. Applied Sterilization Technologies^®^, Swindon, UK) for sterile storage until cell culture.

### 3.1. Cell Culture

The cell line MG63 (ATCC, reference CRL-1427) was selected for in vitro cell culture; this cell line derives from a human osteosarcoma and has osteoblastic characteristics. Cells were cultured in Dulbecco’s modified Eagle medium (DMEM), containing 10% bovine fetal serum (heat inactivated at 56 °C for 30 min.), 2 mM glutamine, 1 mM sodium pyruvate, 1× non-essential amino-acids, penicillin (100 units/mL), and streptomycin (100 µg/mL). Samples were incubated at 37 °C in a 5% CO_2_/95% air atmosphere at 100% relative humidity. The cell culture medium was changed every 2–3 days. When cells reached a density of 85–90% of that showed by a confluence cell culture they were treated with a trypsin-EDTA solution (Gibco, Thermo Fisher Scientific Inc.^®^, Waltham, MA, USA), resuspended in culture media and seeded in new flasks at a 1/5 dilution.

The 120 discs were distributed in 24-well plates. Cells were trypsinized, resuspended in the culture medium as described above, and seeded in the 24-well plates at a density of 5000 cells/cm^2^. To determine the effect of melatonin on MG63 cells, melatonin (Sigma-Aldrich, St. Louis, MO, USA) was added to the culture medium at a final concentration of 50 µM. A 100 mM melatonin stock solution was prepared in 96% ethanol, (suitable for cell culture) and sterilized by filtration using 0.2 µm pore size filters. Aliquots were kept at −20 °C until use. Melatonin was added to cell culture media to a 50 µM final concentration immediately before every change of cell culture media the unused portion of every melatonin aliquot was discarded. As melatonin was dissolved in ethanol, the same volume of ethanol was added to cell culture media used samples cultured in the absence of melatonin to avoid attributing to melatonin any effect that this ethanol concentration might have on cells.

### 3.2. Cell Adhesion, Proliferation and Morphology

To study cell adhesion and proliferation, 24 and 72 h after cell seeding onto the discs, five discs from each experimental group and culture time were selected, both with melatonin present or not in the cell culture media. Discs were washed three times with phosphate-buffered saline (PBS) kept on ice, and cells were fixe for 20 min with 2.5% glutaraldehyde in PBS at room temperature. After 4× PBS washes were stained with 2% eosin (Merck^®^, Darmstadt, Germany) in slightly acidulated with acetic acid water. After 3 min, eosin solution was removed and the samples washed four times with PBS. Samples were then incubated with 0.3 µM 4′,6-diamidino-2-phenylindole (DAPI) in PBS. Samples were kept in this solution until examination under a fluorescence microscope.

Discs were examined under the fluorescence microscope (Leica DM 4000 B, Leica Microsystems^®^, Wetzlar, Germany). Images were taken with a digital camera (DFC 310 XF, Leica Microsystems^®^), using suitable filters and Leica Applications Suite version 4.4.0 software (Leica Microsystems^®^). Images covering each disc whole surface were captured with a 5× objective. To determine the number of cell nuclei per surface unit, the several fluorescence digital images from each disc corresponding to DAPI emission wavelength were combined and the complete image of the surface was reconstructed. Using ImageJ software (National Institutes of Health, Bethesda, MD, USA), the total number of nuclei and surface area of each disc were quantified, calculating the cell density (number of cells/mm^2^).

Cell morphology analysis was performed by SEM (Jeol JSM 6300, Oxford Instruments Ltd.^®^, Abingdon, UK). Twelve discs were randomly selected from those used for cell adhesion and proliferation analysis (four discs per experimental group, two from those disc to which melatonin was added to the cell culture media, and two cultured in the absence of melatonin). Discs with cells already fixed as described above were processed for SEM and kept in 100% ethanol until the moment of critical point drying, when cells were dehydrated in a CO_2_ atmosphere to keep cell morphology. Finally, the discs were coated with a layer of gold and observed under the SEM.

### 3.3. Quantification of Specific mRNA

To study gene expression, MG63 were cultured for one and five weeks, five discs per experimental group and culture time were selected, both with melatonin present or not in the cell culture media. After one and five weeks of cell culture, cell culture plates were put on ice, cell culture media was removed and samples were washed with ice cold Tris-buffered saline (TBS). Cells from each disc were lysed in a total volume of 150 µL of TBS, containing 0.02% Triton-X100 detergent and this volume transferred to 1.5 mL tubes. To favor cell lysis the tubes containing the samples were frozen in liquid nitrogen and quickly unfrozen placing them in a water bath at 37 °C; this process was repeated twice. Total RNA was purified from the cell lysated using TRIzol (Thermo Fisher Scientific, Waltham, MA, USA). The total amount of RNA and its purity were determined using a NanoDrop spectrophotometer. Purified RNA was stored at −80 °C.

A TaqMan Reverse Transcription kit (Thermo Fischer Scientific) was used to obtain the complementary DNA (cDNA) from RNA. 200 ng of total RNA were used and reverse transcription was performed in a final volume of 35 µL, following the manufacturer’s instructions. cDNA samples were stored at −20 °C for later use.

Relative gene expression of the following genes was quantified: alkaline phosphatase (*ALPL*); collagen type 1 α-1 chain (*COL1A-1*); osteocalcin (*OCN*); phosphate-regulating neutral endopeptidase, X-linked (*PHEX*); and osteonectin/secreted protein acidic and rich in cysteine (*SPARC*). Glyceraldehyde-3-phosphate dehydrogenase (*GAPDH*) gene was used as a “housekeeping” gene for normalizing mRNA levels. Quantification of each gene was performed in triplicate, using 384-well plates and the cDNA obtained as described previously. TaqMan gene expression assays specific to each gene were used following recommended conditions by the manufacturer. Quantitative polymerase chain reaction was carried out in a 7900HT Real-Time PCR System thermocycler (Applied Biosystems, Thermo Fisher Scientific).

Using the 7900HT Real-Time PCR System thermocycler analysis software, a cycle threshold (*C*_t_) value was calculated for each amplified sample and the correspondent housekeeping gene *GAPDH* was subtracted to normalize those values, obtaining a d*C*_t_ (Δ*C*_t_) value. The mean value of the three replicas analyzed for each sample was calculated. The level of gene expression showed by cells grown on Group M discs in each experimental condition (cell culture for one or five weeks, with or without melatonin added to the cell culture media) were taken as a reference state. To estimate gene expression relative to this reference state, the mean d*C*_t_ value corresponding to each gene calculated from the five Group M discs was subtracted from the mean d*C*_t_ values, obtaining a dd*C*_t_ (ΔΔ*C*_t_) value. Finally, mean fold change value, the times a gene is expressed in a sample compared to the expression level in a control taken as reference was calculated from the 2^-dd*C*t^ value of the five discs from each study group.

### 3.4. Statistical Analysis

Statistical analysis was performed using IBM SPSS 21.0 statistical software (IBM^®^, New York, NY, USA). The normality of the roughness variable was verified using the Kolmogorov-Smirnov test. Homogeneity of variance in the different groups was evaluated using Levene’s test. The general linear model *F* test and Tamhane’s *T*2 test were applied. The Mann-Whitney non-parametric test was applied to compare cell density and relative gene expression of the different genes between each pair of the study experimental groups. The significance level used for analysis was *p* = 0.05.

## Figures and Tables

**Table 1:** Descriptive results of Ra in the three study groups.

Parameter	Groups			
	Total	Control M	Test E	Test EP
*N*	120	40	40	40
Mean	0.251	0.131	0.266	0.354
Standard deviation	0.111	0.052	0.037	0.088
CI 95%	0.230–0.271	0.115–0.148	0.254–0.278	0.327–0.383
Minimum	0.063	0.063	0.213	0.225
Maximum	0.585	0.359	0.349	0.585
Median	0.256	0.120	0.257	0.327

**Table 2:** Cell density of cells cultured on titanium discs. Descriptive results of mean cell density ± SD by experimental group (discs M, E, and EP), cell culture time (24 and 72 h), and without or with the addition of melatonin to the cell culture media.

Group	Cell Density (Cell Number/mm^2^)			
	Culture Media		Culture Media	
	(without Melatonin)		(with Melatonin)	
	Cell Culture Time		Cell Culture Time	
	24 h	72 h	24 h	72 h
M	84.40 ± 39.52	364.70 ± 61.92	129.51 ± 28.92	611.60 ± 74.07
E	89.28 ± 35.93	306.46 ± 201.83	125.50 ± 35.36	444.37 ± 125.59
EP	53.51 ± 16.43	161.26 ± 79.71	93.10 ± 59.92	109.22 ± 63.89
